# Reading on a smartphone affects sigh generation, brain activity, and comprehension

**DOI:** 10.1038/s41598-022-05605-0

**Published:** 2022-01-31

**Authors:** Motoyasu Honma, Yuri Masaoka, Natsuko Iizuka, Sayaka Wada, Sawa Kamimura, Akira Yoshikawa, Rika Moriya, Shotaro Kamijo, Masahiko Izumizaki

**Affiliations:** Department of Physiology, Showa University School of Medicine, 1-5-8 Hatanodai, Shinagawa-ku, Tokyo, 142-8555 Japan

**Keywords:** Psychology, Human behaviour, Cognitive neuroscience, Reading

## Abstract

Electronic devices have become an indispensable part of our daily lives, while their negative aspects have been reported. One disadvantage is that reading comprehension is reduced when reading from an electronic device; the cause of this deficit in performance is unclear. In this study, we investigated the cause for comprehension decline when reading on a smartphone by simultaneously measuring respiration and brain activity during reading in 34 healthy individuals. We found that, compared to reading on a paper medium, reading on a smartphone elicits fewer sighs, promotes brain overactivity in the prefrontal cortex, and results in reduced comprehension. Furthermore, reading on a smartphone affected sigh frequency but not normal breathing, suggesting that normal breathing and sigh generation are mediated by pathways differentially influenced by the visual environment. A path analysis suggests that the interactive relationship between sigh inhibition and overactivity in the prefrontal cortex causes comprehension decline. These findings provide new insight into the respiration-mediated mechanisms of cognitive function.

## Introduction

In recent years, reading and studying on electronic devices has become more common. Although electronic devices have benefited mankind tremendously, they cause eyestrain and headaches^1,2^ and lead to poor reading comprehension^3,4^. The link between visual environment and cognitive performance has been reported in basic research^5,6^. The decline in comprehension when reading from an electric device might be due to poor concentration levels or different sensory processing circuits, which might be associated with physiological states, including brain and physiological activity levels. Even if the content of the text is the same, reading comprehension may be different depending on the visual context. Because vision has a dominant influence on other senses when it comes to sensory integration or cross-modality^7–9^, visual input might also affect brain state and physiological condition. As such, brain activity and physiological changes likely exist as mediating variables in the relationship between the visual environment and cognitive performance.

We focused on respiration and brain activity as potential mediators. Previous research indicates that overactivity in the brain is associated with poor narrative content comprehension^10–12^. Many studies have reported associations between respiration and various cognitive functions. In these studies, a reciprocal relationship between respiration and cognitive function was found. Respiration is affected by cognitive load^13,14^ or emotion (stress and anxiety)^15^, which alters the depth and rhythm of breathing. Conversely, respiratory patterns affect frontal cortex and hippocampus functions and, consequently, the formation of memories^16–18^. Attention to breathing has also been shown to enhance memory performance^19^. Furthermore, brain activity is entrained to the respiratory cycle^20^. However, no studies have examined the relationship between the visual environment and respiration. According to inter-sensory integration mechanisms, it is known that visual information can alter information from other senses (touch, taste, smell, and auditory). However, information from other senses rarely affects visual sense^7–9^. These findings indicate that vision is dominant over other senses, and it is quite possible that visual environments influence various functions including that of the respiratory system.

Given the findings that the use of digital devices lowers cognitive performance^3,4^, it is probable that the visual environment affects cognitive performance. Regarding cascade processes between the visual environment and cognitive performance, the relationships between the visual environment and brain function^7–9^; brain function and cognitive performance^10–12^; respiration and cognitive performance^16–19^; and respiration and brain function^20^ have been clearly established. However, it is unclear how the visual environment affects respiration and brain function. Therefore, this study investigated the involved mechanism in an exploratory manner. Specifically, we aimed to explore and demonstrate the relationship between the visual environment and respiration and that between the visual environment and brain function to elucidate the phenomenon postulating that the use of digital devices lowers cognitive performance.

If a particular visual environment negatively affects respiratory function and/or brain function, coupled with the interactive relationship between brain and respiratory activity, it is possible that the said process negatively influences cognitive performance (Supplementary Fig. 1). This exploratory study examined the effects of electronic device use on reading comprehension by measuring brain and respiratory activity simultaneously in 34 healthy individuals.

## Results

**Figure 1:**
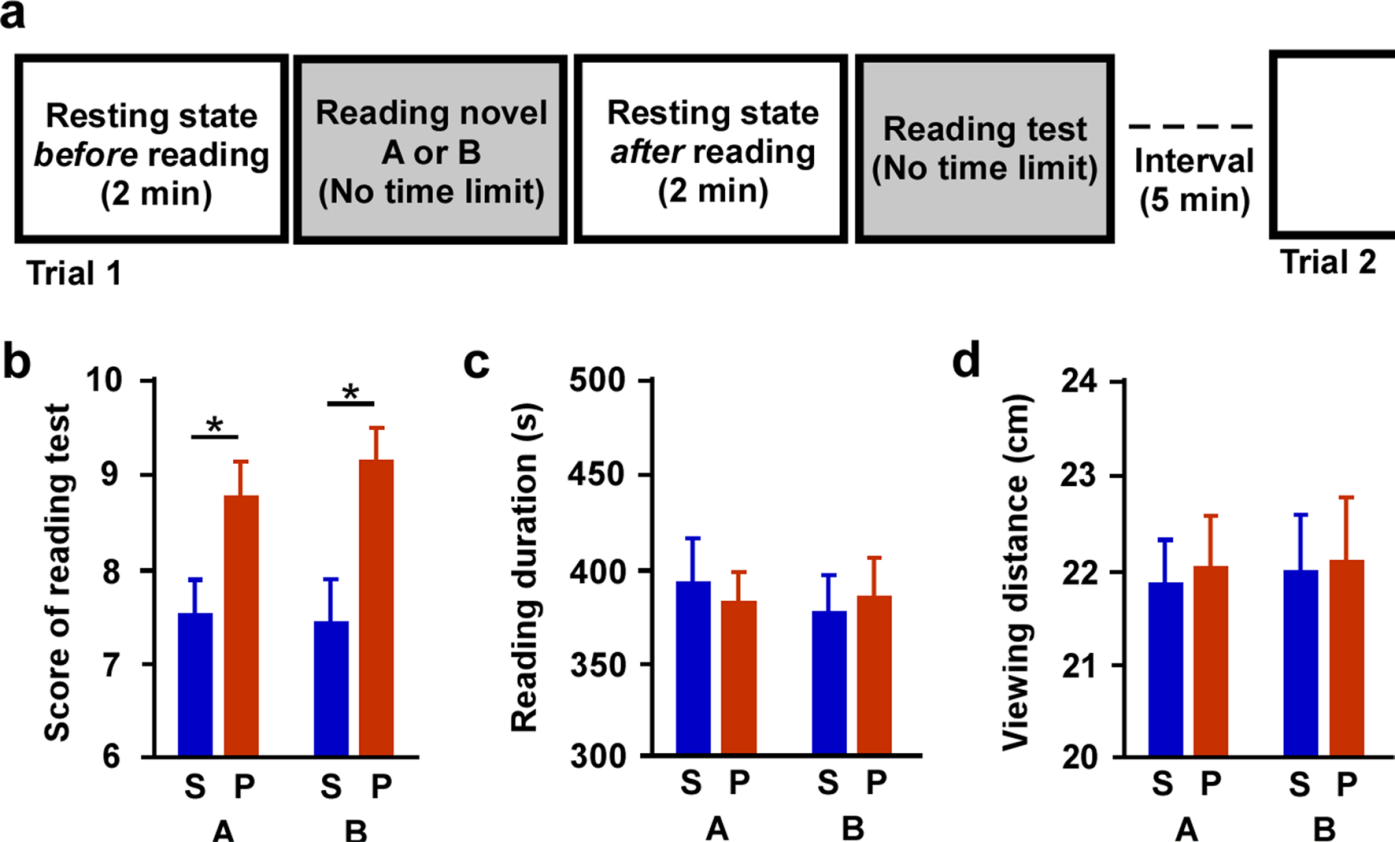
Reading score is decreased by using smartphone compared to paper medium. (**a**) Each trial consisted of four sessions: resting state before reading, reading, resting state after reading, and the reading test. Reading time was unlimited, and two min were spent in the resting state before/after reading. (**b**) For reading test scores, the main effect of medium was significant, while the main effects in novel and the interaction were not. Scores with the smartphone medium were lower than those with the paper medium in sentences from both novel A and B (*P < 0.05). (**c**, **d**) No main or interaction effect was observed for medium or novel in either the duration of reading session or the viewing distance between participants’ eyes and the device. Error bars show standard error of the mean. *S* smartphone, *P* paper, *A* novel A, *B* novel B.

To investigate the causes for reading comprehension decline when using electronic devices, we prepared a repeated-measured (cross-over) design consisting of four conditions involving combinations of two media (smartphone and paper) and two sentences extracted from different texts (novels A and B; see Supplementary Text). One trial consisted of four sessions: resting state before reading, reading, resting state after reading, and reading test (Fig. 1a). Frontal brain activity (two channels) was measured by functional near-infrared spectroscopy (NIRS), while respiratory activity (six indexes) and metabolism pattern (2 indexes) were measured by a respiratory Aeromonitor (see “Methods”).

For the reading score, on cross-over design, repeated-measured analysis of variance (RM-ANOVA) revealed that reading medium affected reading scores (F_1,32_ = 26.445, *P* < 0.0001), while the main effects of novel did not. Post-hoc tests revealed that scores with the paper medium were higher than those with the smartphone in both novels A and B (*P* < 0.05, Fig. 1b). No main or interaction effects of medium or novel were observed for the duration of reading (Fig. 1c) or viewing distance between participants’ eyes and the device (Fig. 1d).

**Figure 2:**
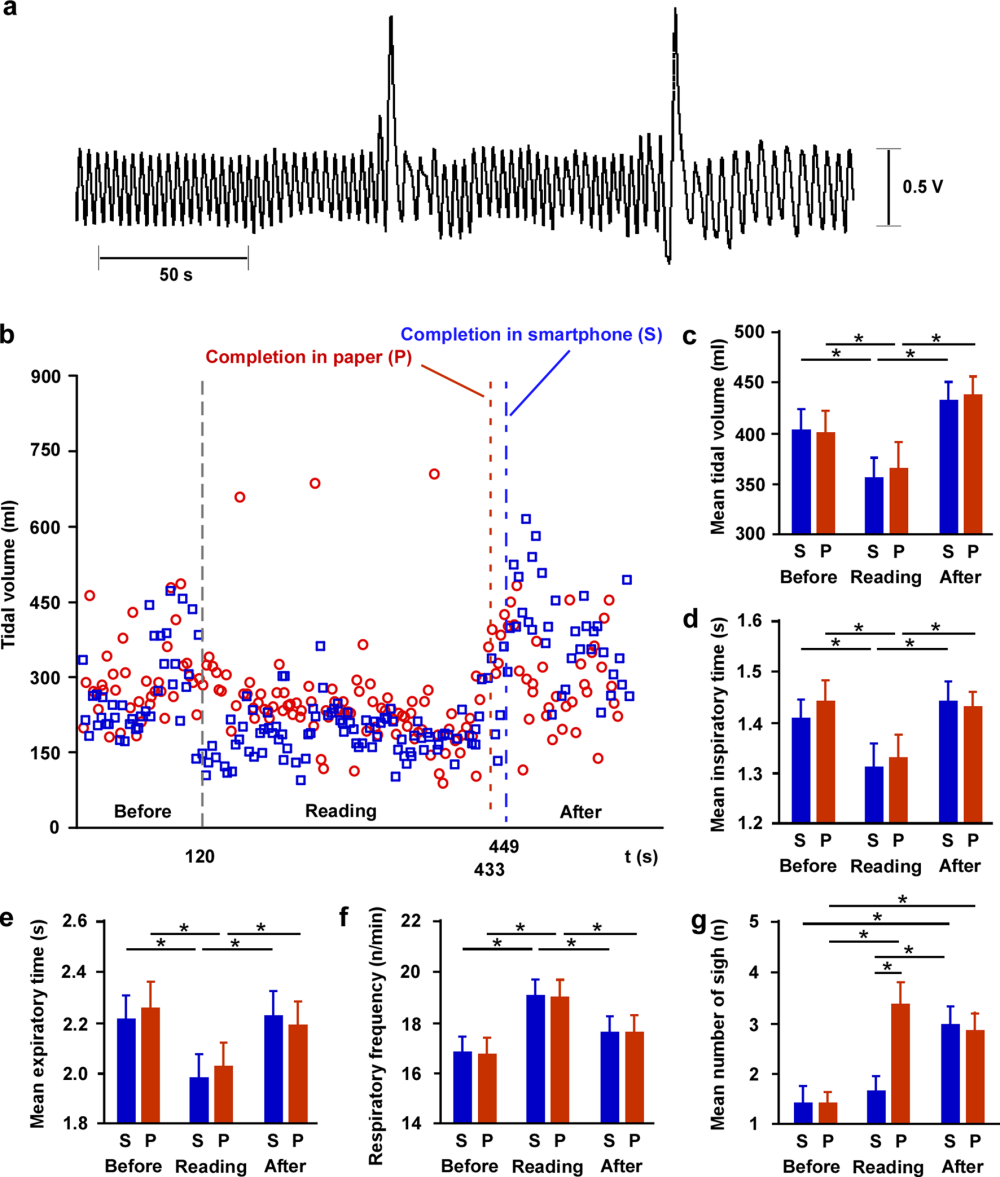
Sighs are inhibited during reading with smartphone compared to paper. (**a**) A typical example of a raw respiration signal from a single participant. (**b**) A representative pattern of the tidal volume and sighs before, during, and after the reading sessions using a smartphone and paper media from a single participant. (**c**–**e**) The mean tidal volume, inspiratory time, and expiratory time reduced during the reading sessions compared to those before and after sessions, regardless of the medium (*P < 0.05). (**f**) The respiratory frequency increased during the reading session compared to that before and after sessions (*P < 0.05). (**g**) The number of sighs increased during the reading session with the paper medium compared to when using a smartphone and increased in the after reading session with both media (*P < 0.05). Error bars show standard error of the mean. *S* smartphone, *P* paper.

Out of six respiratory (Fig. 2a) and two metabolic patterns that we measured, the tidal volume decreased during reading compared to the volume after and before reading sessions, and sighs were increased during reading using a paper medium (Fig. 2b). RM-ANOVA revealed that there was an effect of session, but not of medium, and an interaction for the tidal volume (Fig. 2c, session: F_2,67_ = 8.821, *P* < 0.0001), inspiratory time (Fig. 2d, session: F_2,67_ = 8.639, *P* < 0.0001), expiratory time (Fig. 2e, session: F_2,67_ = 6.212, *P* < 0.0001), respiratory frequency (Fig. 2f, session: F_2,67_ = 5.3622, *P* < 0.0001); there was also an effect of medium, session, and interaction for the number of sighs (Fig. 2g, medium: F_2,67_ = 26.623, *P* < 0.0001; session: F_2,67_ = 6.530, *P* < 0.0001; interaction: F_2,134_ = 9.169, *P* < 0.0001). Post-hoc tests revealed that regardless of the medium, tidal volume, inspiratory time, and expiratory time were reduced during reading compared to those during resting states, while respiratory frequency was increased (all *P* < 0.05). The number of sighs was greater during reading using a paper medium than during reading while using a smartphone (all *P* < 0.05). No changes in minute ventilation, O_2_ consumption, or end-tidal CO_2_ were observed throughout the sessions (Supplementary Fig. 2a–c). In addition, for cases where there were two or more sighs in a trial, the interval duration between sighs was calculated (121 points out of 40 trial) and the mean values were compared in four conditions (smartphone/paper and novels A/B in reading session). The ANOVA indicated no significant differences between conditions (Supplementary Fig. 3).

**Figure 3:**
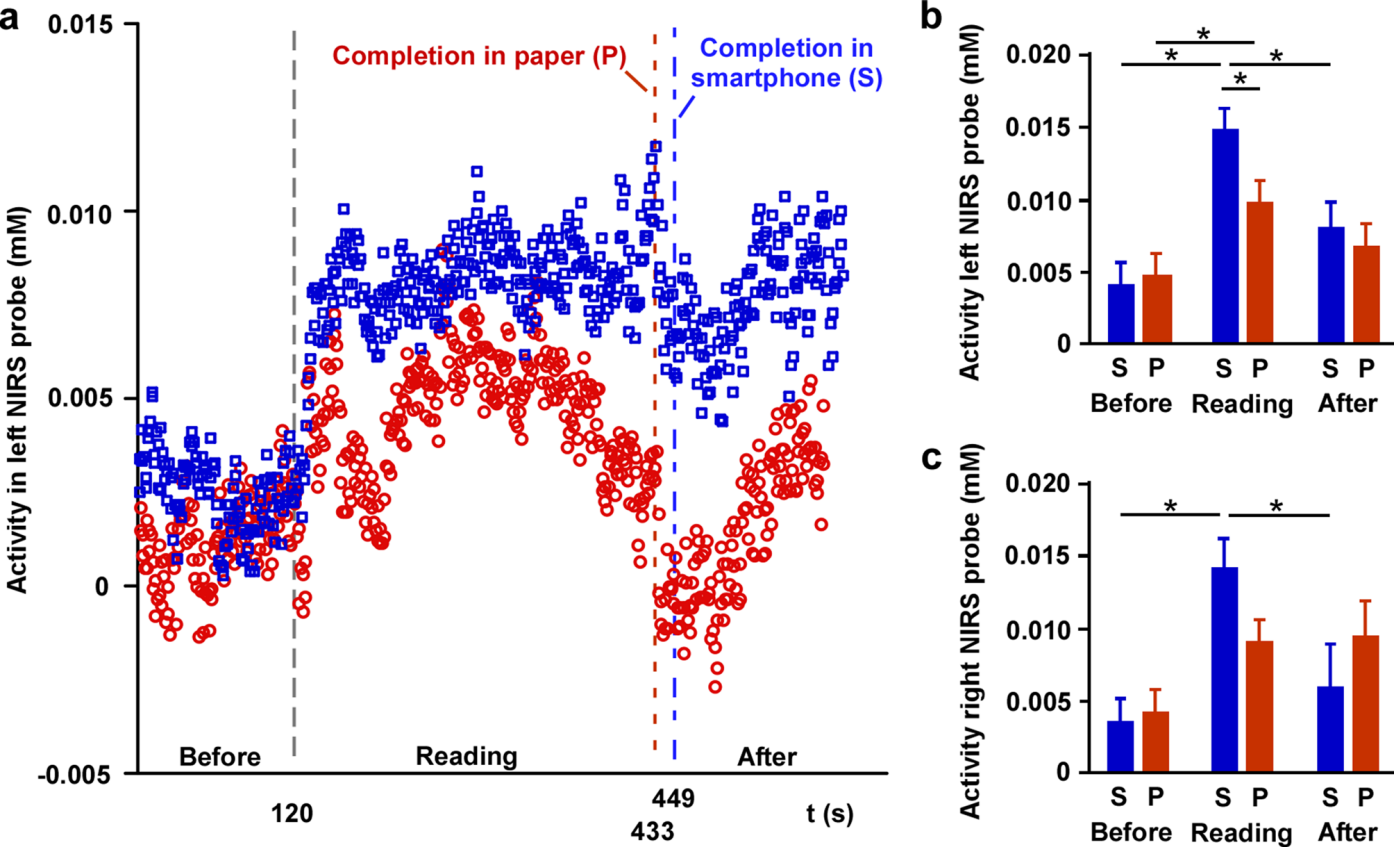
Brain activity around the forehead is increased during reading with a smartphone. (**a**) A representative pattern of functional near-infrared spectroscopy (NIRS) activity on the left probe before, during, and after reading sessions. (**b**, **c**) RM-ANOVA showed that activity recorded with NIRS increased during the reading sessions with a smartphone medium from the left and right probes compared to that before and after reading sessions. The difference between the media was clearly visible in the left probe (*P < 0.05). Error bars show standard error of the mean. *S* smartphone, *P* paper.

We found that activity recorded with NIRS increased during reading compared to that before and after reading sessions and was increased during reading from the smartphone compared to that during reading from the paper medium (Fig. 3a). The RM-ANOVA showed that there was no main effect of medium or interaction in channel 1 (left probe); however, there was a significant main effect of session (F_2,67_ = 17.119, *P* < 0.0001). In channel 2 (right probe), there was no main effect of medium or interaction, while there was a significant main effect of session (F_2,67_ = 10.430, *P* < 0.0001). Post-hoc tests revealed that the activity was increased during reading compared to that during resting states on both the left (*P* < 0.05, Fig. 3b) and right (*P* < 0.05, Fig. 3c) probes. Furthermore, on the left probe, the activity when reading from a smartphone was higher than that when reading from a paper medium (all *P* < 0.05).

**Figure 4:**
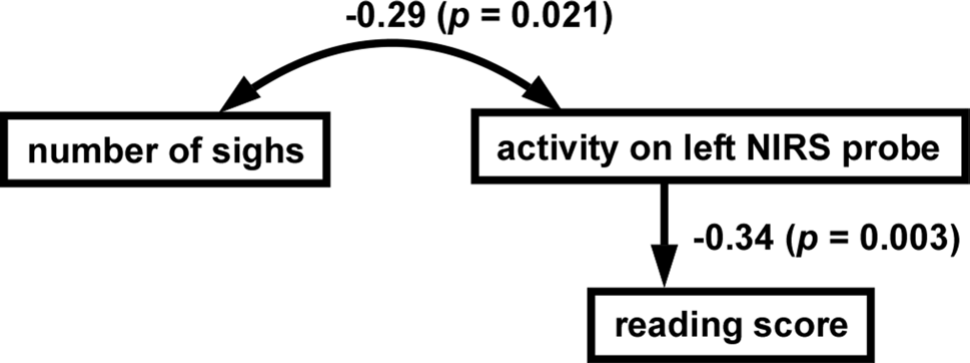
A cascade process in path analysis. Six respiratory indexes (minute ventilation, tidal volume, respiratory frequency, inspiratory time, expiratory time, and number of sighs), two metabolic patterns (O_2_ consumption and End tidal CO_2_), two NIRS indexes (right/left) in the reading sessions, and reading score were set as the observed variables. In the most suitable model (the goodness of fit index = 0.881), there is a covariate relationship between left NIRS channel activity and the number of sighs (*P* = 0.021), and a direct relationship between the right NIRS channel activity and its impact on reading score (*P* = 0.003). Numbers mean standardized path coefficients.

We conducted a path analysis, which identifies causal relationships between variables using path diagrams, to examine a mechanistic route for reading scores. Six respiratory indexes, two metabolic indexes, two NIRS indexes, and reading scores were set as the observed variables. In the most suitable model (Fig. 4, goodness of fit index = 0.881), there was a covariate relationship between left NIRS channel activity and the number of sighs (*P* = 0.021). Moreover, there was a direct relationship between left NIRS channel activity and its impact on reading scores (*P* = 0.003). The increase in left NIRS channel activity correlated with decrease in the number of sighs. In addition, the reading score was low when left NIRS channel activity increased.

## Discussion

The current study replicated the previously reported finding that reading on a smartphone resulted in lower reading performance than reading using a paper medium^3,4^. Respiratory results showed that the average tidal volume decreased and breathing became shallow and fast during reading regardless of the medium, while the number of sighs reduced during reading on a smartphone compared with that during reading using a paper medium, even though the same reading behavior was being performed. For the NIRS data, prefrontal activity in both smartphone and paper was increased during reading session compared to that during a resting state before reading regardless of the medium; furthermore, the activity when using a smartphone was increased compared to that when using a paper medium during reading. Previous studies have indicated that the number of sighs increased with increased cognitive load^14,21^. In our study, the number of sighs increased during cognitive reading activity on a paper medium and decreased when reading on a smartphone. This finding suggests that reading on a smartphone may have caused inhibition of sighs compared to reading on a paper medium. Furthermore, a path analysis suggests that the interactive relationship between sigh inhibition and overactivity in the prefrontal cortex causes the comprehension decline.

If brain function was common with respect to syntactic processing during reading either on paper or on a smartphone, then activity in the prefrontal cortex during smartphone use may be enhanced in the current results. Activity, particularly on the left side, may reflect the predominant activity of the left hemisphere for processing reading^22,23^. Overactivity in the prefrontal cortex has reportedly been associated with poor narrative content comprehension in various subjects^9,10^. The results of the present study showed that overactivity in the prefrontal cortex was observed with smartphone use compared to that when using a paper medium, and the results of lower cognitive performance supported this finding. Generally, increased activity in the prefrontal cortex suggests that the brain underwent a cognitive load. However, overactivity in the prefrontal cortex suggests that the brain was under heavy cognitive load^24^. Regarding reading on a paper medium, moderate cognitive load may have generated sighing (or deep breaths), which appear to restore increased respiratory variability and control of prefrontal brain activity. In contrast, when reading on smartphones, intense cognitive load may have inhibited sigh generation, causing overactivity in the prefrontal cortex. Attention and breathing functions share a common center in the locus coeruleus within the brain^25,26^. Sighing is also associated with increased workload, and breathing variability is restored to a healthy regularity by sighing^27,28^. Sighing may be associated with improved executive functions. These results suggest that a decline in reading comprehension on a smartphone may be caused, at least in part, by reduced sighing and increased prefrontal activity compared to that on a paper medium.

This study examined the effects of the visual environment on cognitive function, and we found an unexpected secondary discovery. Respiratory rhythmogenesis is an emergent property that involves pacemaker neurons within the brainstem and core region of respiratory rhythm formation, called the pre-Bötzinger Complex (preBötC)^29^. Furthermore, it is generally accepted that sigh generation is mediated by a different mechanism than normal breathing. While a classical study reported that bilateral vagus nerve transection abolished sighing^30^, a different study observed that two hours after bilateral vagus nerve transection^31^, spontaneous sighing still occurred in anesthetized cats, leading to some controversy regarding the role of the vagus nerve in sighing. A recent report suggested that sighing is regulated by two parallel bombesin-like neuropeptide pathways in mice, neuromedin B and gastrin-releasing peptide, which mediate signaling between respiratory control centers^32^. The neurons project to the preBötC, and activation of the preBötC receptor increases sighing by approximately 10 times, whereas inhibition of the receptor abolishes sighing. Alternatively, manipulation of the receptors had no effect on normal breathing, confirming that normal breathing and sighing are mediated by different pathways. Based on this study, our findings suggest that visual environment of reading affects sigh generation but not normal breathing.

What causes the decrease in sighing incidence using a smartphone? One of the possible causes of this is the blue light emitted by smartphones. Intrinsically photosensitive retinal ganglion cells (ipRGCs) that respond to blue light project to various brain regions such as the peri-habenular nucleus, which modulate arousal and learning^33^. Blue light also causes forced sustained attention^34^. Furthermore, a recent epidemiological study reported a link between exposure to blue light from smartphones and increased arousal and anxiety^35^. The decreased sighing and brain overactivity in smartphone use may be caused by sustained cognitive load in ipRGC activity due to blue light exposure. Further research is needed to examine the effect of blue light on respiration and brain activity.

This study provides a new perspective on the relationship between the visual environment and cognitive performance, based on the results of path analysis (Supplementary Fig. 5). Regarding reading on a paper medium, moderate cognitive load may generate sighs (or deep breaths) and appears to restore respiratory variability and control of prefrontal brain activity. In contrast, reading on smartphones may require sustained task attention^34^, and acute cognitive load may inhibit the generation of sighs, causing overactivity in the prefrontal cortex. Sighing has been found to be associated with various cognitive functions^13,27,28^, and may reset respiratory variability^36,37^. This reset may also be associated with improved executive functions^14^.

The current study has several limitations. First, our experiment did not entail any measurement of subjective cognitive load. Based on the differences in the number of sighs and brain activity between reading on smartphones and paper media, it is highly likely that there might have been a difference in cognitive load as well. In future, it is necessary to assess cognitive load indices and examine the relationship between breathing and brain activity. Second, we did not control the movements when turning pages or pointing movements to maintain the focus of attention on the text. These bodily movements may have had some influence on the present index. In the future, such physical limitations should be taken into consideration.

The results of this study suggest that reduced reading comprehension on smartphone devices may be caused by reduced sighing and overactivity of the prefrontal cortex, although the effect on electronic devices other than smartphones has yet to be confirmed. Recent reports indicate that the use of smartphones and other electronic devices has been increasing due to pandemic-related lockdowns, and there are indications that this is negatively influencing sleep and physical activity^38,39^. The relationships among visual environment, respiration/brain activities, and cognitive performance detected in this study may indicate one of the negative effects of electronic device use on the human body. If the negative effects of smartphones are true, it may be beneficial to take deep breaths while reading since sighs, whether voluntary or involuntary, regulate disordered breathing^36^.

## Materials and methods

### Participants

This study was approved by the ethics committee of Showa University School of Medicine and conducted according to the principles of the Declaration of Helsinki (trial identifier number: 2179). Thirty-four Japanese university students provided written informed consent prior to the experiments. All participants were right-hand dominant and had no history of neurological or psychiatric disease (20 females; mean age = 20.4, standard deviation = 0.8). Participants had normal vision with/without correction.

### Experimental design and setting

In this study, cognitive performance was evaluated by scores on the reading comprehension test at the end of one trial. Physiological conditions included respiration (respiratory pattern, number of sighs, and metabolic pattern) and brain functions (prefrontal activity by NIRS). The factor of visual environment consisted of smartphone and paper media. To examine the influence of using a smartphone for reading, we set up four conditions involving a combination of two media (smartphone and paper) and two sentences (novels A and B; see Supplementary Text) (Fig. 1a). Participants took part in two randomly conducted trials for each condition to avoid duplication of medium and novel conditions (for example, if the first trial was novel A on a smartphone, the second trial was novel B on paper) (Supplementary Fig. 4). All conditions were counterbalanced, based on the condition that medium type and novel type did not overlap. A trial consisted of four sessions: resting state before reading, during reading, resting state after reading, and reading test. Participants were instructed to sit and their torso and arms were secured. They were asked to read the allocated novel on the allocated medium during the reading session. During resting state sessions, participants were asked to open their eyes and look at a wall. Subsequently, they took a reading test consisting of ten questions related to the contents of the novels. They were also asked to breathe through their nose during all sessions. There was no limit on reading time, and two min was spent in the resting state before/after reading. At the end of the experiment, participants were asked whether they had previously read the novels used in the experiment. All participants responded that they had never read the novels.

The observation distance was determined by the convenience of the participants, and the experimenter measured the distance between the device and the participants’ eyes. The factors of medium and novel had no influence on reading time or viewing distance. Each novel originated from a passage of one of two novels written by the same author, Haruki Murakami (novel A: Norwegian Wood, 1987, 3060 Japanese characters; or novel B: Colorless Tsukuru Tazaki and His Years of Pilgrimage, 2013, 3067 Japanese characters). The panel size of the smartphone was 5.0 inches (resolution pixel: height: 1920, width: 1080), and the text size was 0.85 degrees. The text size on the paper medium was identical to that on the smartphone. In addition, the weight (148 g) and outer frame (height: 144 mm, width: 72 mm, thickness: 8.6 mm) of the paper medium were also identical to those of the smartphone.

Participants wore a band on their forehead to measure brain activity and a mask around their mouth and nose to measure respiration. NIRS (Hb13-2, ASTEM, Kanagawa, Japan) consisted of two probes for the measurement of brain activity, which took measurements from the left and right prefrontal cortex^40,41^. The NIRS calculated oxygenated hemoglobin concentration [mM] on the forehead. The probe consisted of an LED that emits light and a photodiode that receives the light transmitted through the body. The amount of oxidized and deoxidized hemoglobin was estimated by quantifying the spatial slope of light scattering and transmission in body tissues.

An air-cushioned face mask (AMA104, Minato Medical Science, Osaka, Japan) was attached to a headband (KBN0226, Minato Medical Science, Osaka, Japan) and a transducer (hot-wire flowmeter, Minato Medical Science, Japan: diameter and length of respiratory tubing: 5 mm, 205 cm) was connected to a respiratory monitor (Aeromonitor AE-280, Minato Medical Science, Japan) for the measurement of respiratory patterns and metabolism^42^. The measurement of breath by breath was calculated with software (AT Window, Minato Medical Science, Japan) and raw respiratory signals were simultaneously measured by another software (Lab Chart, AD Instruments, New Zealand) via an AD converter (PowerLab, AD Instruments, New Zealand). The respiratory monitor calculated six indexes of respiratory pattern: tidal volume (depth of breathing, [ml]), respiratory frequency (number of breaths per minute, [n/min]), inspiratory time (duration to inhale breath, [s]), expiratory time (duration to exhale breath, [s]), minute ventilation (ventilation rate per minute, [l]), number of sighs (deep breathing, [n]), and two indexes of the metabolic pattern (O_2_ consumption [ml], End-tidal CO_2_ [%]). A sigh was defined as at least twice the average tidal volume for each session^43^. The interval duration between sighs was analyzed and the analysis was limited to cases in which two or more sighs during reading occurred within a single trial.

### Statistics

RM-ANOVA and post-hoc *t*-tests were performed to test the main effects and interactions of medium (smartphone and paper) and novel sentence (novels A and B) on the scores for the reading test, reading duration, viewing distance, and interval time between sighs. They were also performed for medium (smartphone and paper) and session conditions (before, during, and after reading) on respiration (tidal volume, inspiratory time, expiratory time, respiratory frequency, minute ventilation, End tidal CO_2_, O_2_ consumption, and number of sighs) and brain function (right and left activities of the prefrontal cortex). All tests were two-tailed. Results are presented as mean ± standard error of the mean. JMP Pro 16.0 (SAS Institute, Inc) was used for RM-ANOVA. The statistical significance criterion was defined as adjusted *P* < 0.05 with Bonferroni correction. Relationships among the 6 respiration indexes, 2 metabolism indexes, 2 NIRS indexes, and the comprehension score were calculated by path analysis. The goodness of fit of models was determined with the root-mean square of approximation. AMOS 27.0 was used for path analysis.

## Supplementary Information

Supplementary Information.

## Data Availability

Supplementary information is provided with the paper to support the current results. Analyses used in this study are largely standard approaches for this type of data. Other data that support the findings of this study are available from the corresponding author upon request.
