# Supplementary material for: Reading on a smartphone affects sigh generation, brain activity, and comprehension
Source: Sci Rep. 2022 Jan 31;12:1589. doi: 10.1038/s41598-022-05605-0 (PMC8803971; doi:10.1038/s41598-022-05605-0)
Supplement: Supplementary file 1 — Supplementary Information. [file 41598_2022_5605_MOESM1_ESM.pdf]

Supplementary Information for

Reading on a smartphone affects sigh generation, brain activity, and comprehension

Motoyasu Honma[1*], Yuri Masaoka[1], Natsuko Iizuka[1], Sayaka Wada[1], Sawa Kamimura[1], Akira Yoshikawa[1], Rika Moriya[1], Shotaro Kamijo[1], Masahiko Izumizaki[1].

[1] Department of Physiology, Showa University School of Medicine, 1-5-8 Hatanodai, Shinagawa-ku, Tokyo 142-8555, Japan.

*Correspondence to: Motoyasu Honma

Department of Physiology, Showa University School of Medicine

1-5-8 Hatanodai, Shinagawa-ku, Tokyo 142-8555, Japan

Tel: +81 3 3784 8781, Fax: +81 3 3784 1936, Email: mhonma@med.showa-u.ac.jp

Supplementary Figs. 1-5

Supplementary Text

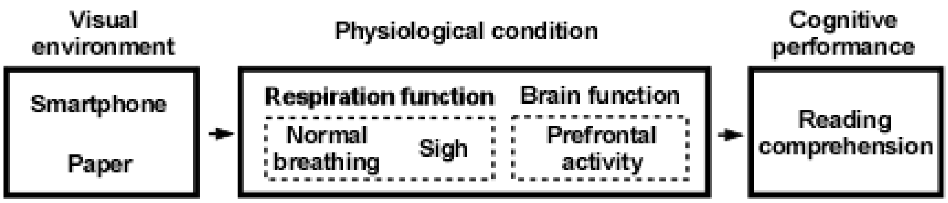

Supplementary Fig. 1 | Concept of study. Brain activity and physiological changes should exist as mediating variables in the relationship between the visual environment and cognitive function.

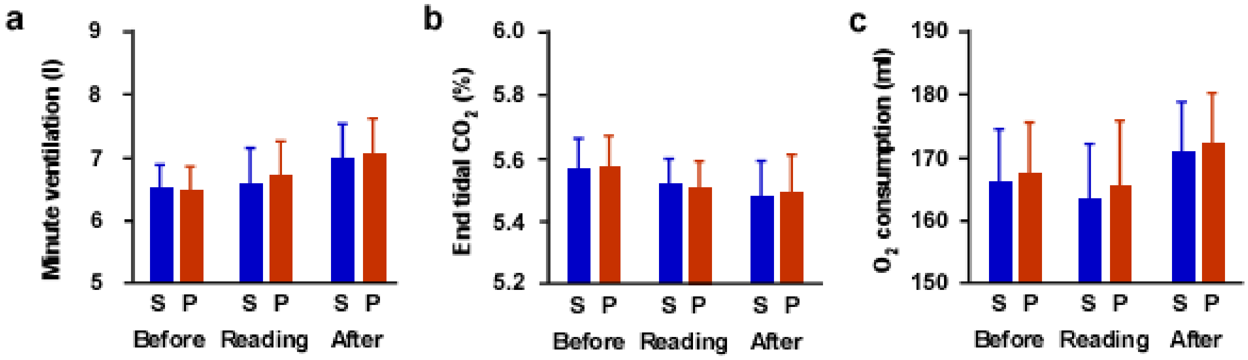

Supplementary Fig. 2 | Effect of medium and session on minute ventilation and metabolic patterns. RM-ANOVA showed that no main or interaction effects of medium and session were observed in **(a)** minute ventilation (medium: $F_{1,67} = 0.079$, $P = 0.779$; session: $F_{2,134} = 0.453$, $P = 0.503$), **(b)** O_2 consumption (medium: $F_{1,67} = 0.110$, $P = 0.741$; session: $F_{2,134} = 0.545$, $P = 0.463$), or **(c)** end-tidal CO_2 (medium: $F_{1,67} = 0.022$, $P = 0.882$; session: $F_{2,134} = 1.785$, $P = 0.187$).

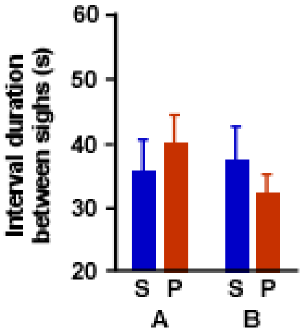

Supplementary Fig. 3 | Effect of medium and session on interval duration between sighs. An interval duration between sighs was analyzed, and the analysis was limited to cases in which two or more sighs during reading occurred within a single trial (total 121 points of 40 trials). ANOVA showed that no main or interaction effects of medium and session were observed in interval duration between sighs (medium: $F_{1,36} = 0.610$, $P = 0.806$; novel: $F_{1,36} = 1.551$, $P = 0.221$; interaction: $F_{1,36} = 1.990$, $P = 0.167$).

	Trial 1	Trial 2
Order 1	Smartphone<br>Novel A	Paper<br>Novel B
Order 2	Smartphone<br>Novel B	Paper<br>Novel A
Order 3	Paper<br>Novel A	Smartphone<br>Novel B
Order 4	Paper<br>Novel B	Smartphone<br>Novel A

Supplementary Fig. 4 | Each experiment was performed two times per participant. Experimenters determined the order of the combination of two media (smartphone and paper) and two novels (Novels A and B) by random order (four possible orders). Each participant participated in any one of the four order types.

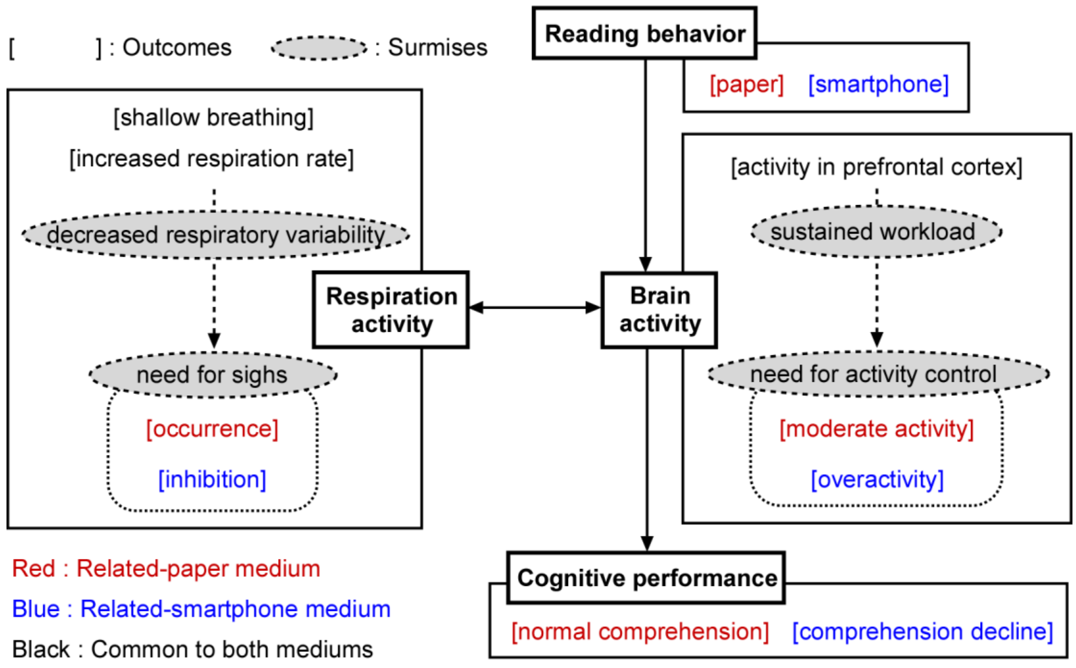

Supplementary Fig. 5 | Possible mechanism of reading processing with interactive relationship between respiratory and brain activity. A relation diagram based on reading behavior, brain activity, respiration activity, and cognitive performance. The terms in parentheses are outcomes from the current experiment. The terms enclosed in a dotted circle are surmises from previous researches. Terms in red text are paper-related, in blue text are smartphone-related, and in black text are matters that are common to both media.

Supplementary Text

<u>Questions and answer options for the reading test</u>

Novel A (Norwegian Wood (Japanese version), pages 1-6, Haruki Murakami, 1987; 3060 Japanese characters)

Q1: How old was the protagonist?

A: 35 B: 36 C: 37 D: 38 E: 39

Q2: What was the model number of the Boeing of the plane the protagonist was flying in?

A: 737 B: 747 C: 757 D: 767 E: 777

Q3: What was the name of the airport where the protagonist arrived?

A: Hamburg B: Frankfurt C: Munich D: Zurich E: Vienna

Q4: In what month did the story take place?

A: August B: September C: October D: November E: December

Q5: What confused the protagonist?

A: Voices of the passengers B: The sound of the air-conditioning C: atmospheric pressure

D: background music E: The Captain's Voice

Q6: What was the nationality of the cabin attendant?

A: French B: American (USA) C: Italian D: English E: German

Q7: In what language did the protagonist respond to the cabin attendant?

A: English B: German C: French D: Italian E: Japanese

Q8: What was the landscape that the protagonist remembered?

A: Pasture B: Grassland C: Urban center D: Sea E: Forest

Q9: How old did the protagonist remember himself??

A: Seventeen B: Eighteen C: Nineteen D: Twenty E: Twenty-one

Q10: What was the name of the woman the protagonist remembered?

A: Junko B: Misaki C: Yuki D: Takako E: Naoko

Novel B (Colorless Tsukuru Tazaki and His Years of Pilgrimage (Japanese version), pages 1-6, Haruki Murakami, 2013; 3067 Japanese characters)

Q1: How old was the protagonist?

A: 19 B: 20 C: 21 D: 22 E: 23

Q2: What was the protagonist's position?

A: High school student B: Unemployed C: Vocational school student D: University student E: Office worker

Q3: How long did the protagonist's longing for death last?

A: Three months B: Six months C: Nine months D: Twelve months

E: Eighteen months

Q4: What was the protagonist's routine?

A: Walking B: Reading newspaper C: Listening to the radio D: Listening to music E: Watching TV

Q5: How many times a week did the protagonist do laundry?

A: One B: Two C: Three D: Four E: Five

Q6: What type of alcohol did the protagonist drink?

A: Beer B: Brandy C: Wine D: Sake E: Whisky

Q7: How many best friends did the protagonist have in high school?

A: Zero B: One C: Two D: Three E: Four

Q8: What city was the protagonist's high school near?

A: Yokohama B: Chiba C: Shizuoka D: Nagoya E: Fukuoka

Q9: What was the subject of the protagonist's high school assignment?

A: English B: Math C: Science D: National language E: Social study

Q10: How many days did the protagonist attend summer camp?

A: One B: Two C: Three D: Four E: Five
